# Variation in Thermal Stability among Respiratory Syncytial Virus Clinical Isolates under Non-Freezing Conditions

**DOI:** 10.3390/v14040679

**Published:** 2022-03-25

**Authors:** Yuki Kitai, Ko Sato, Kazuya Shirato, Suguru Ohmiya, Oshi Watanabe, Tomoko Kisu, Reiko Ota, Makoto Takeda, Kazuyoshi Kawakami, Hidekazu Nishimura

**Affiliations:** 1 Virus Research Center, Clinical Research Division, Sendai Medical Center, Miyagino 2-11-12, Miyagino-ku, Sendai 983-8520, Japan; kitai.yuuki.hj@mail.hosp.go.jp (Y.K.); oomiya.suguru.qj@mail.hosp.go.jp (S.O.); watanabe.oshi.zr@mail.hosp.go.jp (O.W.); kisu.tomoko.es@mail.hosp.go.jp (T.K.); oota.reiko.gw@mail.hosp.go.jp (R.O.); 2 Department of Medical Microbiology, Mycology and Immunology, Tohoku University Graduate School of Medicine, Sendai 980-0872, Japan; ko-sato@med.tohoku.ac.jp (K.S.); kawakami@med.tohoku.ac.jp (K.K.); 3 Department of Intelligent Network for Infection Control, Tohoku University Graduate School of Medicine, Sendai 980-0872, Japan; 4 Department of Virology III, National Institute of Infectious Disease, Tokyo 208-0011, Japan; shirato@nih.go.jp (K.S.); mtakeda@nih.go.jp (M.T.)

**Keywords:** respiratory syncytial virus, storage temperature, thermal stability, virus inactivation, virus isolation

## Abstract

Virus isolates are not only useful for diagnosing infections, e.g., respiratory syncytial virus (RSV), but can also facilitate many aspects of practical viral studies such as analyses of antigenicity and the action mechanisms of antivirals, among others. We have been isolating RSV from clinical specimens from patients with respiratory symptoms every year since our first isolation of RSV in 1964, and isolation rates have varied considerably over the years. As collected clinical specimens are conventionally stored in a refrigerator from collection to inoculation into cells, we hypothesized that certain storage conditions or associated factors might account for these differences. Hence, we evaluated the thermal stability of a total of 64 viruses isolated from 1998 to 2018 upon storage at 4 °C and 20 °C for a defined duration. Interestingly, and contrary to our current understanding, 22 strains (34%) showed a greater loss of viability upon short-term storage at 4 °C than at 20 °C. Thirty-seven strains (57%) showed an almost equal loss, and only five strains (8%) were more stable at 4 °C than at 20 °C. This finding warrants reconsideration of the temperature for the temporary storage of clinical samples for RSV isolation.

## 1. Introduction

Respiratory syncytial virus (RSV) is usually a pathogen of the upper respiratory tract, but it can sometimes cause severe bronchiolitis and pneumonia in infants, young children, immunocompromised patients, and the elderly [1,2,3]. RSV is categorized into two subgroups, A and B, based on the amino acid sequence of the G protein, with the prevalent subgroup differing in successive epidemic seasons [4,5,6,7].

Virus isolation is an important tool for diagnosis because of its high specificity, but the number of facilities that isolate viruses has been decreasing. Most clinical settings use PCR or viral antigen assays that are immunochromatography-based rapid antigen-detection kits, probably because of the challenges associated with viral isolation and the long time required for obtaining results. However, virus isolation not only remains relevant for research purposes, such as analyses of antigenicity, susceptibility to antivirals, and immunogenicity, but also affords greater specificity compared with genetic or antigen detection, all of which lead to a precise diagnosis of an actual infection.

Since the first isolation of RSV in 1964 in a clinical research laboratory, we have continued to isolate RSV from clinical specimens for diagnosis, surveillance, and research on viral infections [8,9,10,11]. During this long period of RSV isolation, we experienced yearly fluctuations in the isolation rate of tested clinical specimens, i.e., lower rates of isolation irrespective of the prevalent subgroup(s). Traditionally, in the optimal procedure for short-term storage, each clinical specimen is collected and stored for a certain period, from one to several days, at 4 °C until inoculation into cultured cells for viral isolation. RSV is known to be labile to a higher temperature, namely, room temperature (20 °C), as well as the procedure of freeze-thawing [12,13]. In contrast to this current understanding of RSV and based on a scrutiny of our laboratory records, we suspected that storage at 4 °C might affect the viability of some viral strains. Hence, we used a series of RSV isolates to investigate the effects on infectivity after storage at 4 °C and compared them after storage at 20 °C. We show that although some strains retained infectivity despite several days of storage at 4 °C, others lost this characteristic, and some strains more rapidly lost their viability upon storage at 4 °C than at 20 °C. This report describes these interesting phenomena in detail.

## 2. Materials and Methods

### 2.1. Ethical Considerations

All experimental procedures were approved by the Ethics Committee of the Sendai Medical Center (SMC), Sendai, Japan. All RSV isolates used in this study were obtained from pediatric patients with respiratory symptoms who visited the pediatric clinic of the SMC between 1998 and 2018.

### 2.2. Specimens for Virus Isolation and Surveillance of RSV Infection in the Community

Nasal swabs, nasal aspirates, and throat swabs were collected from patients with respiratory symptoms who presented to clinics and hospitals in Sendai, Japan, between 1998 and 2018, and they were stored in 3 mL of the transport medium, which is minimum essential medium (MEM; Sigma-Aldrich, St. Louis, MO, USA) containing 0.5% gelatin (Junsei Chemical, Tokyo, Japan), 500 units/mL of penicillin G, and 500 µg/mL of streptomycin (Meiji, Tokyo, Japan). Between 2009 and 2018, samples were subjected to the antigen-detection test using a point of care testing (POCT) immunochromatography rapid test (ImunoAce RSV Neo, Tauns, Shizuoka, Japan), which is routine practice in clinical settings, followed by storage at 4 °C in a refrigerator until virus isolation was attempted. A total of 182 clinical specimens that were positive in the antigen-screening test were subjected to the genetic analyses by RT-PCR for confirmation and further study.

### 2.3. Viruses Used for Experiments

Between 1998 and 2018 in Sendai, Japan, 64 RSV strains isolated according to the virus isolation protocol were used in the thermal stability assay [9]. Briefly, collected samples in the transport medium were centrifugated at 3000 rpm at 4 °C for 15 min. The supernatant was inoculated in the HEp-2 cell line seeded in 96-well microplates (Corning, NY, USA) followed by plate centrifugation for 30 min at 2000 rpm at room temperature. After centrifugation, the plates were incubated in a 5% CO_2_ incubator at 34 °C for a month. Cytopathic effect (CPE) was checked twice a week. Virus isolation was attempted without blind passage. Each virus strain was propagated by culturing it 1–5 times in the HEp-2 cell line, and it was harvested with the culture media as virus stocks when the CPE had spread throughout the cell layer. Viral titer was calculated based on 50% tissue culture infectious dose (TCID_50_). Virus stocks were snap frozen in a dry ice-ethanol bath and stored at −80 °C until use.

### 2.4. Cell Culture

For RSV isolation and experiments, HEp-2 cells available in our laboratory were cultured in MEM containing 5% calf serum (CS; Thermo Fisher Scientific, Waltham, MA, USA), 1% fetal bovine serum (FBS; Thermo Fisher Scientific), 1.7% glucose (TERUMO, Tokyo, Japan), 100 unit/mL of penicillin G, and 100 µg/mL of streptomycin.

### 2.5. RNA Extraction, RT-LAMP, and RT-Quantitaitive PCR (RT-qPCR)

Viral RNA was extracted from samples using the QIAamp Viral RNA Mini Kit (Qiagen, Limburg, The Netherlands) according to manufacturer’s protocol. Subgrouping of the RSV strains was performed based on reverse transcription-loop-mediated isothermal amplification (RT-LAMP) [14] with the Loopamp^®^ RT-LAMP Kit (Eiken, Tokyo, Japan), RSV-A or -B specific primers, or RT-qPCR [15] (Table 1).

RT-qPCR was performed to quantify viral load, as previously described [16]. RNA extracted from clinical specimens was amplified using primers and probes specific for each subgroup (Table 1).

### 2.6. Thermal Stability Assay

Viral titers from stocks were adjusted to 1.0 × 10^5^ TCID_50_/_mL_, stored at 4 °C or 20 °C for a defined duration for the experiment, and subjected to an infectivity assay that determined TCID_50_ unit using serial ten-fold dilutions of the sample and HEp-2 cells cultured in a 96-well microplate. After inoculation of each dilutant, the plate was centrifuged at 2000 rpm for 30 min followed by incubation in a 5% CO_2_ incubator at 34 °C for 5 days. Subsequently, development of CPE was observed, and the viral titer was calculated according to the Leed and Mench method [17,18].

### 2.7. Statistical Analysis

Data were analyzed using JMP^®^Pro 11.2.0 software (SAS Institute Japan, Tokyo, Japan). Differences between groups were examined for statistical significance using the Man–Whitney U test. A *p* value less than 0.05 was considered statistically significant.

## 3. Results

### 3.1. Yearly Changes in RSV Isolation Rate

**Figure 1:**
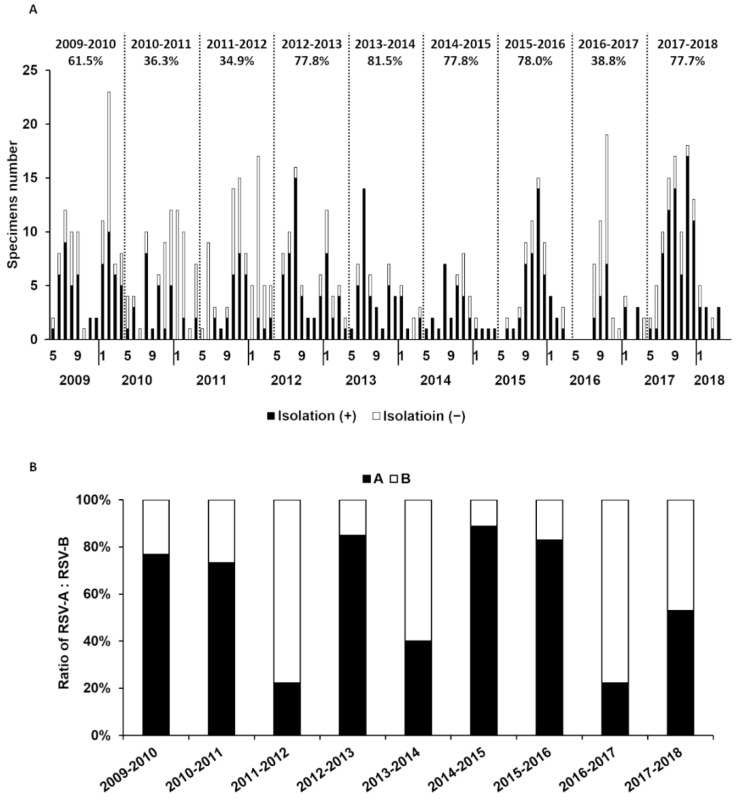
Yearly changes in RSV isolation rate for 10 years from 2009 with prevalent subgroups. (**A**) The isolation rate of RSV in each epidemic season was calculated and is shown above the graph. The isolation rate is expressed as the number of isolation-positive specimens divided by all RSV- antigen-positive specimens. Solid and open bars indicate isolated and non-isolated sample numbers, respectively. (**B**) RSV subgroups were detected by RT-LAMP method. Ratios of solid bars and open bars indicate the percentage of the subgroups.

The virus isolation started to increase from around June, peaked before the end of the year, and declined until around May the following year, forming a yearly cycle of the epidemic season (Figure 1A). RSV positivity was defined based on the results of the viral antigen test, and the RSV isolation rate of the season was calculated as the number of successful isolations divided by the number of RSV-positive cases for that season; the relevant time period ranged from 2009 to 2018. The isolation rate fluctuated during this period and was found to be low in some seasons (2010–2011, 2011–2012, 2016–2017) (Figure 1A). When the isolation rate was calculated after the subgrouping of RSV-positive cases, no correlation was identified between subgroups and isolation rates (Figure 1B).

**Figure 2:**
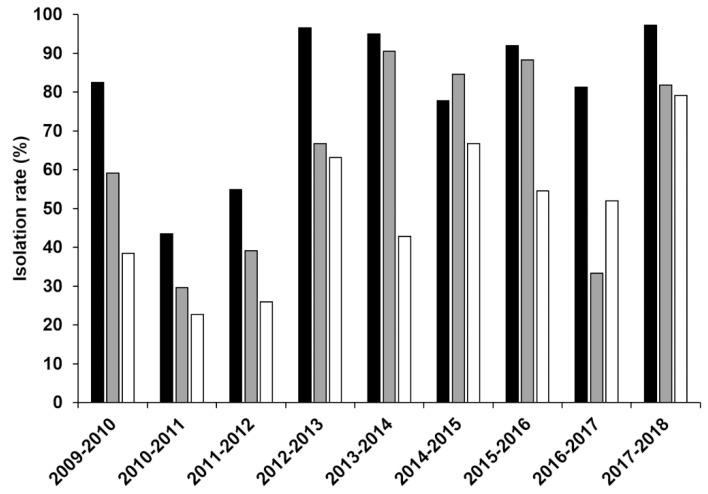
Isolation rates of RSV after storage at 4 °C. The isolation rate of RSV by storage periods is shown. Solid bar denotes isolation rate of specimens stored at 4 °C before inoculation into HEp-2 cells on the same day of specimen collection. Gray colored and open bars indicate the isolation rate of specimens stored for 1 day or 2 days after collection, respectively, at 4 °C.

A detailed analysis of the records showed that RSV could not be isolated in many cases even though RSV was detected by viral antigen tests (Figure 1A) and that specimens inoculated on days other than the collection day were potentially responsible for the low isolation rate (Figure 2). Furthermore, isolation rates gradually decreased day-by-day after specimen collection in some seasons (2009–2010, 2011–2012, 2013–2014, 2014–2015, 2015–2016) but showed a significant decrease only the next day in other seasons (2010–2011, 2012–2013, 2016–2017) (Figure 2), suggesting that the loss of viral viability occurred within one day when stored at 4 °C. On the other hand, the virus could be isolated from some samples even after a longer storage period of more than two days in those seasons.

**Figure 3:**
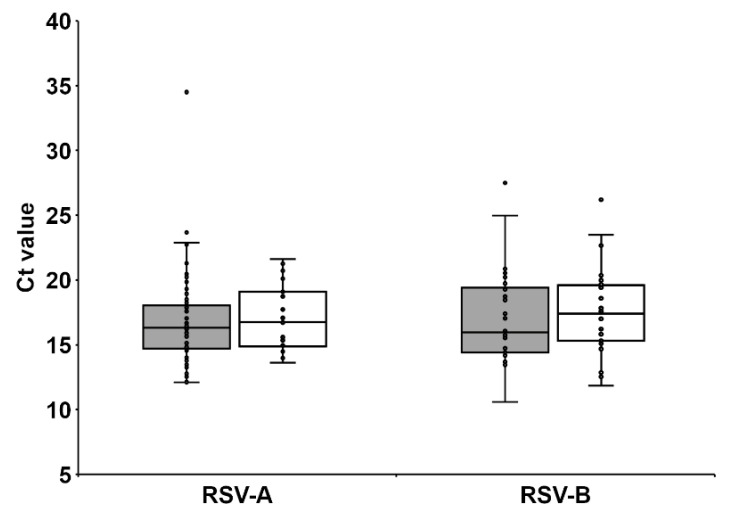
Relative comparison of RSV load in clinical specimens by quantitative real-time PCR. RNA was extracted from specimens (140 µL) and amplified by real-time PCR. Ct values of the samples were plotted. Gray and open squares show Ct value of isolation positive and negative specimens, respectively.

Because a possible reason for virus isolation failure could be lower viral load in those specimens, we genetically quantified RSV load in the clinical specimens using RT-qPCR and found no significant difference in viral load between specimen groups from which the virus could be isolated and those from which it could not (Figure 3). These observations strongly indicate that storage conditions before inoculation are key factors that determine the success of virus isolation. Furthermore, the resistance to the loss of viability may be different for each RSV strain.

### 3.2. Stability of RSV at 4 °C

**Figure 4:**
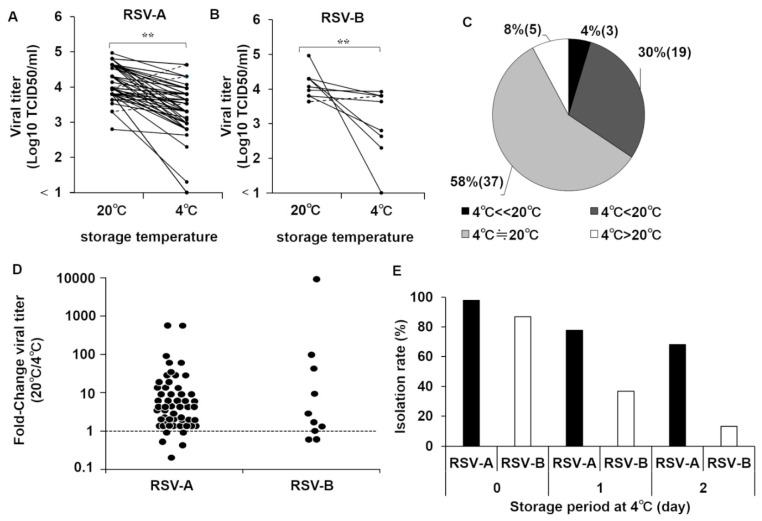
Comparison of viability of RSV isolates at 4 °C and 20 °C. The infective titers of RSV samples were adjusted to 1.0 × 10^5^ TCID50/mL, stored at 4 °C or 20 °C for 24 h, and titer was measured again by the TCID50 assay. (**A**,**B**) The titers after storage at 4 °C or 20 °C are plotted. Each isolate is identified by a line between the storage temperatures tested. Solid line indicates that viral titer was higher when stored at 20 °C, whereas broken line indicates vice versa. **, *p* < 0.05. (**C**) Percentage of thermal stability of RSV isolates. Solid gray and light gray areas denote percentage (and number) of RSV strains that were more stable at room temperature than at 4 °C. Open area shows the percentage of RSV strains stable at 4 °C compared with room temperature. (**D**) Titer after storage at 20 °C was divided by that at 4 °C, and the ratio was plotted. Broken line denotes identical stability upon storage at 4 °C and 20 °C. (**E**) Isolation rates based on length of storage period of clinical specimens are shown by RSV subgroups.

Next, we analyzed the stability of RSV isolates at 4 °C and compared it with the results produced at 20 °C (room temperature). The reduction in viral titers during storage at these temperatures was investigated using viral stocks available in our laboratory, i.e., RSV isolated from clinical specimens and stored at −80 °C after isolation. Viruses kept at 20 °C and 4 °C showed a slight decrease in infective titer compared with samples just recovered from −80 °C storage (1.0 × 10⁵ TCID_50_/_mL_). However, to our surprise, many samples kept at 20 °C showed titers higher than those kept at 4 °C (Figure 4A–D), irrespective of when they were isolated; specifically, only 5 strains (8%) had a higher number of folds at 4 °C than at 20 °C (Figure 4C). Subgroup B viruses appeared to be more labile at 4 °C than subgroup A viruses based on the data regarding the difference between isolation rates in clinical specimens by lengths of storage periods, but there were exceptions (Figure 4E). Viral load in the 4 °C-labile and non-labile groups was quantified using RT-qPCR, which showed similar values (data not shown). At the extremes, three strains, RSV/Sendai 1369/98 (group A), RSV/Sendai 2006/98 (group A), and RSV/Sendai 851/13 (group B), were completely inactivated within 24 h of storage at 4 °C but stable at 20 °C.

**Figure 5:**
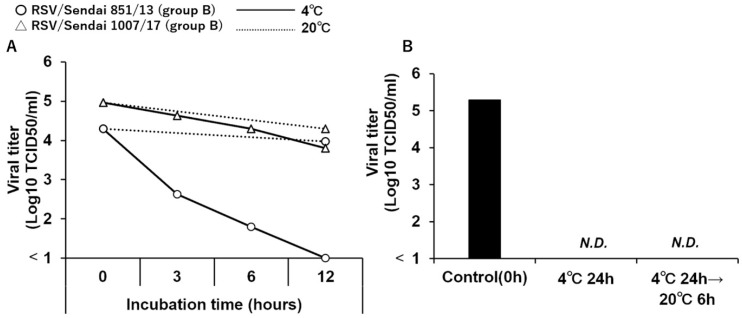
Time course of reduction in viability during storage at 4 °C. (**A**) Infectivity titers of viral seeds of RSV/Sendai 851/13 (group B) and RSV/Sendai 1007/17 (group B) were adjusted to 1.0 × 10⁵ TCID50/mL, and their aliquots were stored at 4 °C for 0 h, 3 h, 6 h, or 12 h. After storage, infectivity titer was measured by the TCID50 assay. Circles show unstable samples at 4 °C, whereas triangles denote stable samples. (**B**) The viral seed of RSV/Sendai 851/13 (group B), with infectivity titer adjusted to 1.0 × 10^5^ TCID50/mL, was stored at 4 °C for 24 h, transferred to 20 °C for 6 h, and infectivity titer was measured again by the TCID50 assay.

The viability of the representative strain, RSV/Sendai 851/13 (group B), started to decrease in a few hours and was completely lost by 12 h; this was in clear contrast to the RSV/Sendai 1007/17 (group B) strain, which only showed a slight decrease during the same time period. (Figure 5A).

Next, to evaluate the potential recovery of the reduced viability when the virus was kept at room temperature, samples that showed a reduction in viability when kept at 4 °C were then kept at 20 °C for 6 h. However, this transfer did not lead to the recovery of viability, indicating that temperature-induced inactivation was an irreversible phenomenon (Figure 5B).

## 4. Discussion

We showed that some RSV strains lost infectivity when kept at 4 °C for short periods of time, and most strains could maintain their viral titer when they were kept at 20 °C compared with when they were kept at 4 °C. Generally, non-freezing low temperatures (4 °C) during short-term storage are thought to not result in the loss of infectivity for viruses; this is especially true for RSV as it is broadly believed to be susceptible to room temperature and to freeze-thawing [12,13]. Conventionally, it has been the norm to maintain RSV at a low temperature during manipulation. It must be noted here that these studies predominantly used laboratory strains of RSV, such as the Long and A2 strains, which were isolated more than 50 years ago and have been passaged many times in cultured cells; this could have altered their original characteristics and rendered them different from those of wild isolates subjected to only a few passages, such as those used in this study.

Indeed, Gucht et al. reported that their RSV isolates between 2016 and 2018 showed different characteristics compared with laboratory strains, such as low levels of viral replication in the culture, high fusion capacity, and low viability at certain temperatures (4 °C, 32 °C, and 37 °C) [19].

A limitation of this study is that the viruses included in the experiments were those that were deemed isolation positive via the appearance of CPE. Thus, this might not reflect the temperature sensitivity at 4 °C and 20 °C of viruses that could not cause CPE. 

Our results indicate the possibility that the storage of clinical specimens at 4 °C can lead to a failure of RSV isolation in some specimens and a fall in the isolation rate in some seasons. The poor condition of the HEp-2 cells might be the cause of the lower isolation rate of RSV in those seasons. However, the HEp-2 cells were not out of condition because the isolation rate of adenoviruses isolated from the same cells was stable across all seasons (data not shown). Although we acknowledge that it is ideal for clinical specimens to be inoculated into cultured cells as quickly as possible after collection, we also acknowledge that it might not always be possible in daily practice at the clinical laboratory.

Given this, a room temperature of around 20 °C, rather than at 4 °C, might be preferable for temporary storage prior to RSV inoculation into cultured cells. Nevertheless, as some strains might lose their infectivity when kept at room temperature, for optimal efficiency of RSV isolation, it may be necessary to divide specimens for storage at both temperatures prior to inoculation and subsequent isolation.

Mechanistically, inactivation at 4 °C rather than at 20 °C is very interesting, and future research using the 4 °C-labile strains identified in this study could address aspects of the viral replication cycle in the cell that are affected by virus storage at this temperature. It is known that steric changes in protein structure occur not only upon heating but also upon cooling, albeit the latter is infrequent. Additionally, as Stobart et al. have suggested that the five amino acid residues on the F gene are involved in the thermal stability of RSV, we posit that the function or functions of the fusion protein that exists on the virus envelope may also be of significance [20]. However, all these amino acid residues were the same between the 4 °C-sensitive and non-sensitive strains. Thus, further studies are required to understand the inactivation mechanism at 4 °C. Furthermore, our study helped in choosing an appropriate temperature to avoid unnecessary inactivation during the temporary storage for the distribution of a vaccine in clinical settings when the live RSV vaccine becomes available; it also helped in the investigation of the mechanism or mechanisms that lead to inactivation based on storage temperatures. Our study may contribute to the development of stable, live vaccines that cannot be affected by storage temperatures in their supply-chain.

## Figures and Tables

**Table 1:** Primers and probes used in this study.

Assay	Function	Sequence (5′-3′)
RT-qPCR	Forward primer	GGCAAATATGGAAACATACGTGAA
	Reverse primer	TCTTTTTCTAGGACATTGTAYTGAACAG
	Probe	FAM-CTGTGTATGTGGAGCCTTCGTGAAGCT-BHQ
LAMP (RSV-A)	RSAM-F3	GGGGCAAATATGGAAACATACGT
	RSAM-B3	GAAGGTCCATTGGGTGTG
	RSAM-FIP	AGGGTCATCGTCTTTTTCTAAGACATTTTTTCACGAAGGCTCCACAT
	RSAM-BIP	ATCACTTACAATATGGGTGCCCTTTTGTATGTTGACATTAGCTAGTTCT
	RSAM-LF	TTGTATTGAACAGCAGCTGTGT
	RSAM-LB	ATGCCAGCAGATTTACTTATA
LAMP (RSV-B)	RSBM-F3	GGGCAAATATGGAAACATACG
	RSBM-B3	CCTTTGGGCGTAGAGATC
	RSBM-FIP	GTTAGTGATGCAGGATCATCATCTTTTTTTGAACAAGCTTCACGAAGG
	RSBM-BIP	TATGGGTGCCTATGTTCCAGTTTTTGCTTCACTAGTATGTTGATGCT
	RSBM-LF	GTACTGAACAGCTGCTGTGTAT
	RSBM-LB	GCAGACTTGCTCATAAAAGAACTTG
	RSAM-F3	GGGGCAAATATGGAAACATACGT
Subgrouping	Forward primer	GGAAACATACGTGAACAAGCTTCA
	Reverse primer (A)	CATCGTCTTTTTCTAGGACATTGTATT
	Reverse primer (B)	TCATCATCTTTTTCTAGAACATTGTACTGA
	Probe	FAM-TGTGTATGTGGAGCCTT-MGB

## Data Availability

Not applicable.
